# *N*′-(3-Eth­oxy-2-hydroxy­benzyl­idene)-4-hydr­oxy-3-methoxy­benzohydrazide monohydrate

**DOI:** 10.1107/S1600536809033236

**Published:** 2009-09-05

**Authors:** Jiu-Fu Lu, Yue-Fei Bai, Suo-Tian Min, Hong-Guang Ge, Xiao-Hui Ji

**Affiliations:** a School of Chemistry and Environmental Science, Shaanxi University of Technology, Hanzhong 723000, People’s Republic of China; b School of Pharmaceutical Engineering, Shenyang Pharmaceutical University, Shenyang 110016, People’s Republic of China

## Abstract

In the title compound, C_17_H_18_N_2_O_5_·H_2_O, the dihedral angle between the two aromatic rings is 7.86 (7)° and an intra­molecular O—H⋯N hydrogen bond is observed. In the crystal structure, mol­ecules are linked into a three-dimensional network by inter­molecular O—H⋯O and N—H⋯O hydrogen bonds.

## Related literature

For related structures, see: Lu *et al.* (2008*a* ▶,*b* ▶,*c* ▶); Abdul Alhadi *et al.* (2009 ▶); Mohd Lair *et al.* (2009 ▶); Narayana *et al.* (2007 ▶). For bond-length data, see: Allen *et al.* (1987 ▶).
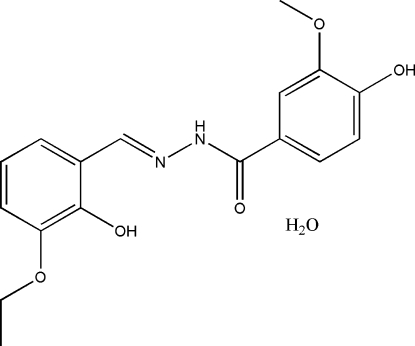


## Experimental

### 

#### Crystal data

C_17_H_18_N_2_O_5_·H_2_O
*M* *_r_* = 348.35
Monoclinic, 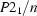

*a* = 9.4063 (11) Å
*b* = 10.0598 (12) Å
*c* = 17.667 (2) Å
β = 93.702 (2)°
*V* = 1668.3 (3) Å^3^
*Z* = 4
Mo *K*α radiation
μ = 0.11 mm^−1^
*T* = 298 K
0.23 × 0.20 × 0.20 mm

#### Data collection

Bruker APEXII CCD area-detector diffractometer
Absorption correction: multi-scan (*SADABS*; Sheldrick, 2004 ▶) *T*_min_ = 0.976, *T*_max_ = 0.979
9554 measured reflections
3606 independent reflections
2530 reflections with *I* > 2σ(*I*)
*R*_int_ = 0.022

#### Refinement

*R*[*F* ^2^ > 2σ(*F* ^2^)] = 0.041
*wR*(*F* ^2^) = 0.111
*S* = 1.05
3606 reflections
239 parameters
4 restraints
H atoms treated by a mixture of independent and constrained refinement
Δρ_max_ = 0.14 e Å^−3^
Δρ_min_ = −0.18 e Å^−3^

### 

Data collection: *APEX2* (Bruker, 2004 ▶); cell refinement: *SAINT* (Bruker, 2004 ▶); data reduction: *SAINT*; program(s) used to solve structure: *SHELXS97* (Sheldrick, 2008 ▶); program(s) used to refine structure: *SHELXL97* (Sheldrick, 2008 ▶); molecular graphics: *SHELXTL* (Sheldrick, 2008 ▶); software used to prepare material for publication: *SHELXTL*.

## Supplementary Material

Crystal structure: contains datablocks global, I. DOI: 10.1107/S1600536809033236/ci2887sup1.cif

Structure factors: contains datablocks I. DOI: 10.1107/S1600536809033236/ci2887Isup2.hkl

Additional supplementary materials:  crystallographic information; 3D view; checkCIF report

## Figures and Tables

**Table 1:** Hydrogen-bond geometry (Å, °)

*D*—H⋯*A*	*D*—H	H⋯*A*	*D*⋯*A*	*D*—H⋯*A*
O1—H1⋯N1	0.82	1.94	2.6529 (16)	144
O5—H5⋯O6^i^	0.82	1.81	2.6177 (17)	170
O6—H6*A*⋯O3^ii^	0.84 (1)	1.96 (1)	2.7908 (17)	176 (2)
O6—H6*B*⋯O1^iii^	0.84 (1)	2.08 (1)	2.8714 (18)	157 (2)
N2—H2⋯O4^iv^	0.89 (1)	2.54 (2)	3.1181 (17)	123 (2)
N2—H2⋯O5^iv^	0.89 (1)	2.19 (1)	3.0496 (18)	163 (2)

Symmetry codes: (i) 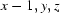; (ii) 
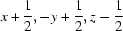
; (iii) 
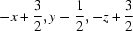
; (iv) 
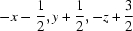
.

**Fig. 1.**
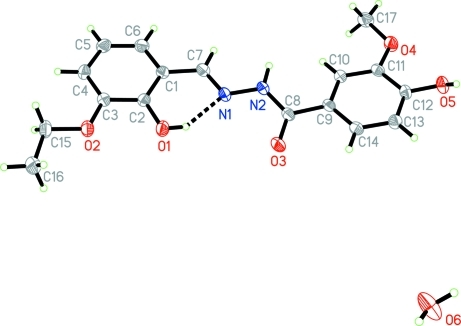
The molecular structure of the title compound, showing 30% probability displacement ellipsoids and the atom-numbering scheme. The dashed line indicates a hydrogen bond.

**Fig. 2.**
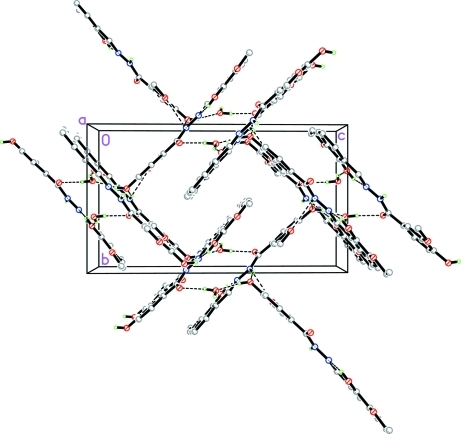
The crystal packing of the title compound, viewed along the a axis. Hydrogen bonds are shown as dashed lines. H atoms not involved in hydrogen bonding have been omitted for clarity.

## supplementary crystallographic information

### Comment

Schiff bases and their metal complexes have received much attention in recent years. As part of our investigation on crystal structures of Schiff bases derived from the condensation of aldehydes with benzohydrazides (Lu *et al.*,  2008*a*,b,c), we report herein the crystal structure of the title new Schiff base compound.

The asymmetric unit of the title compound (Fig. 1), consists of a Schiff base molecule and a water molecule of crystallization. The bond lengths have normal values (Allen *et al.*,  1987), and are comparable to those observed in similar compounds (Abdul Alhadi *et al.*, 2009; Mohd Lair *et al.*, 2009; Narayana *et al.*,  2007). The dihedral angle between the two aromatic rings is 7.86 (7)°, indicating that they are approximately coplanar. The methoxy and ethoxy groups are coplanar with the attached rings [C17—O4—C11—C10 = 4.6 (2)°,  C15—O2—C3—C4 = -2.6 (2)° and C3—O2—C15—C16 = 177.98 (14)]. An intramolecular O—H···N hydrogen bond is observed (Table 1 and Fig. 1).

In the crystal structure, molecules are linked into a three-dimensional network (Fig. 2) by intermolecular O—H···O and N—H···O hydrogen bonds (Table 1).

### Experimental

The title compound was prepared by the Schiff base condensation of 2-hydroxy-3-ethoxybenzaldehyde (0.1 mol) and 4-hydroxy-3-methoxybenzohydrazide (0.1 mmol) in 95% ethanol (50 ml). The excess ethanol was removed by distillation. The colourless solid obtained was filtered and washed with ethanol. Single crystals suitable for X-ray diffraction were obtained by slow evaporation of a 95% ethanol solution at room temperature.

### Refinement

The imino and water H atoms were located in a difference map and refined with N-H, O-H, and H···H distance restraints of 0.90 (1), 0.85 (1) and 1.37 (2) Å, respectively. Other H atoms were positioned geometrically (C-H = 0.93-0.97 Å, O-H = 0.82 Å) and refined using a riding model, with U_iso_(H) = 1.2U_eq_(C) and 1.5U_eq_(C_methyl_ and O).

### Crystal data

**Table:**

C_17_H_18_N_2_O_5_·H_2_O	*F*(000) = 736
*M**_r_* = 348.35	*D*_x_ = 1.387 Mg m^−^^3^
Monoclinic, *P*2_1_/*n*	Mo *K*α radiation, λ = 0.71073 Å
Hall symbol: -P 2yn	Cell parameters from 2358 reflections
*a* = 9.4063 (11) Å	θ = 2.3–24.5°
*b* = 10.0598 (12) Å	µ = 0.11 mm^−^^1^
*c* = 17.667 (2) Å	*T* = 298 K
β = 93.702 (2)°	Block, colourless
*V* = 1668.3 (3) Å^3^	0.23 × 0.20 × 0.20 mm
*Z* = 4	

### Data collection

**Table:**

Bruker APEXII CCD area-detector diffractometer	3606 independent reflections
Radiation source: fine-focus sealed tube	2530 reflections with *I* > 2σ(*I*)
graphite	*R*_int_ = 0.022
ω scans	θ_max_ = 27.0°, θ_min_ = 2.3°
Absorption correction: multi-scan (*SADABS*; Sheldrick, 2004)	*h* = −12→6
*T*_min_ = 0.976, *T*_max_ = 0.979	*k* = −12→12
9554 measured reflections	*l* = −19→22

### Refinement

**Table:**

Refinement on *F*^2^	Primary atom site location: structure-invariant direct methods
Least-squares matrix: full	Secondary atom site location: difference Fourier map
*R*[*F*^2^ > 2σ(*F*^2^)] = 0.041	Hydrogen site location: inferred from neighbouring sites
*wR*(*F*^2^) = 0.111	H atoms treated by a mixture of independent and constrained refinement
*S* = 1.05	*w* = 1/[σ^2^(*F*_o_^2^) + (0.0506*P*)^2^ + 0.153*P*] where *P* = (*F*_o_^2^ + 2*F*_c_^2^)/3
3606 reflections	(Δ/σ)_max_ = 0.001
239 parameters	Δρ_max_ = 0.14 e Å^−^^3^
4 restraints	Δρ_min_ = −0.18 e Å^−^^3^

### Special details

**Table:**

Geometry. All esds (except the esd in the dihedral angle between two l.s. planes) are estimated using the full covariance matrix. The cell esds are taken into account individually in the estimation of esds in distances, angles and torsion angles; correlations between esds in cell parameters are only used when they are defined by crystal symmetry. An approximate (isotropic) treatment of cell esds is used for estimating esds involving l.s. planes.
Refinement. Refinement of F^2^ against ALL reflections. The weighted R-factor wR and goodness of fit S are based on F^2^, conventional R-factors R are based on F, with F set to zero for negative F^2^. The threshold expression of F^2^ > 2sigma(F^2^) is used only for calculating R-factors(gt) etc. and is not relevant to the choice of reflections for refinement. R-factors based on F^2^ are statistically about twice as large as those based on F, and R- factors based on ALL data will be even larger.

### Fractional atomic coordinates and isotropic or equivalent isotropic displacement parameters (Å^2^)

**Table:**

	*x*	*y*	*z*	*U*_iso_*/*U*_eq_	
N1	0.10205 (14)	0.58113 (13)	0.92228 (7)	0.0430 (3)	
N2	0.02746 (14)	0.49793 (13)	0.87165 (7)	0.0439 (3)	
O1	0.33352 (11)	0.69470 (12)	0.98717 (7)	0.0540 (3)	
H1	0.2897	0.6377	0.9621	0.081*	
O2	0.44363 (11)	0.88030 (11)	1.07376 (6)	0.0500 (3)	
O3	0.22665 (12)	0.38467 (12)	0.84788 (6)	0.0540 (3)	
O4	−0.35636 (11)	0.22118 (12)	0.71623 (6)	0.0519 (3)	
O5	−0.21352 (12)	0.07248 (12)	0.62606 (6)	0.0495 (3)	
H5	−0.1832	0.0803	0.5838	0.074*	
O6	0.87031 (16)	0.12628 (18)	0.49098 (7)	0.0831 (5)	
C1	0.09270 (16)	0.76123 (14)	1.00984 (8)	0.0386 (4)	
C2	0.23985 (16)	0.77422 (14)	1.02122 (8)	0.0381 (3)	
C3	0.29798 (17)	0.87517 (15)	1.06851 (8)	0.0401 (4)	
C4	0.20833 (19)	0.95870 (16)	1.10488 (9)	0.0461 (4)	
H4	0.2463	1.0260	1.1361	0.055*	
C5	0.06202 (19)	0.94300 (17)	1.09513 (9)	0.0491 (4)	
H5A	0.0024	0.9986	1.1207	0.059*	
C6	0.00461 (18)	0.84627 (16)	1.04808 (9)	0.0455 (4)	
H6	−0.0938	0.8371	1.0415	0.055*	
C7	0.02778 (17)	0.66469 (15)	0.95732 (8)	0.0432 (4)	
H7	−0.0708	0.6636	0.9489	0.052*	
C8	0.09772 (16)	0.40175 (15)	0.83586 (8)	0.0384 (4)	
C9	0.01099 (15)	0.31662 (14)	0.78123 (8)	0.0351 (3)	
C10	−0.13784 (16)	0.31459 (14)	0.77666 (8)	0.0365 (3)	
H10	−0.1878	0.3686	0.8084	0.044*	
C11	−0.21102 (15)	0.23219 (15)	0.72488 (8)	0.0366 (3)	
C12	−0.13654 (17)	0.15245 (14)	0.67632 (8)	0.0377 (3)	
C13	0.01011 (17)	0.15321 (15)	0.68193 (8)	0.0420 (4)	
H13	0.0602	0.0985	0.6506	0.050*	
C14	0.08347 (16)	0.23511 (15)	0.73412 (8)	0.0397 (4)	
H14	0.1825	0.2351	0.7374	0.048*	
C15	0.50926 (19)	0.98456 (17)	1.11853 (11)	0.0572 (5)	
H15A	0.4796	0.9801	1.1701	0.069*	
H15B	0.4817	1.0705	1.0974	0.069*	
C16	0.66733 (19)	0.9667 (2)	1.11801 (12)	0.0642 (5)	
H16A	0.6931	0.8805	1.1379	0.096*	
H16B	0.7144	1.0340	1.1489	0.096*	
H16C	0.6958	0.9740	1.0670	0.096*	
C17	−0.44022 (17)	0.30663 (19)	0.75946 (10)	0.0555 (5)	
H17A	−0.4195	0.2898	0.8125	0.083*	
H17B	−0.5394	0.2902	0.7466	0.083*	
H17C	−0.4184	0.3975	0.7484	0.083*	
H2	−0.0640 (12)	0.519 (2)	0.8616 (11)	0.080*	
H6A	0.8271 (18)	0.119 (2)	0.4483 (7)	0.080*	
H6B	0.9512 (13)	0.161 (2)	0.4869 (11)	0.080*	

### Atomic displacement parameters (Å^2^)

**Table:**

	*U*^11^	*U*^22^	*U*^33^	*U*^12^	*U*^13^	*U*^23^
N1	0.0430 (8)	0.0417 (7)	0.0424 (7)	−0.0028 (6)	−0.0102 (6)	−0.0046 (6)
N2	0.0378 (7)	0.0425 (7)	0.0495 (7)	−0.0006 (6)	−0.0130 (6)	−0.0076 (6)
O1	0.0423 (7)	0.0555 (7)	0.0638 (8)	0.0032 (6)	−0.0008 (6)	−0.0259 (6)
O2	0.0426 (7)	0.0509 (7)	0.0560 (7)	−0.0028 (5)	−0.0007 (5)	−0.0188 (5)
O3	0.0348 (6)	0.0661 (8)	0.0591 (7)	0.0020 (6)	−0.0115 (5)	−0.0130 (6)
O4	0.0321 (6)	0.0615 (7)	0.0616 (7)	−0.0057 (5)	−0.0012 (5)	−0.0146 (6)
O5	0.0477 (7)	0.0577 (7)	0.0426 (6)	−0.0144 (6)	0.0003 (5)	−0.0110 (5)
O6	0.0632 (9)	0.1350 (14)	0.0491 (7)	−0.0436 (9)	−0.0107 (7)	0.0188 (8)
C1	0.0404 (9)	0.0381 (8)	0.0367 (8)	0.0013 (7)	−0.0030 (7)	0.0019 (6)
C2	0.0421 (9)	0.0359 (8)	0.0360 (7)	0.0038 (7)	0.0012 (7)	−0.0027 (6)
C3	0.0393 (9)	0.0415 (8)	0.0391 (8)	0.0004 (7)	−0.0014 (7)	−0.0028 (7)
C4	0.0549 (11)	0.0411 (9)	0.0420 (8)	0.0016 (8)	−0.0001 (8)	−0.0104 (7)
C5	0.0500 (10)	0.0490 (10)	0.0488 (9)	0.0113 (8)	0.0060 (8)	−0.0058 (7)
C6	0.0416 (9)	0.0481 (9)	0.0465 (9)	0.0043 (8)	0.0008 (7)	0.0004 (7)
C7	0.0396 (9)	0.0448 (9)	0.0442 (9)	0.0001 (7)	−0.0061 (7)	0.0000 (7)
C8	0.0367 (9)	0.0412 (9)	0.0362 (8)	−0.0015 (7)	−0.0055 (7)	0.0024 (6)
C9	0.0349 (8)	0.0366 (8)	0.0328 (7)	−0.0019 (7)	−0.0047 (6)	0.0039 (6)
C10	0.0364 (8)	0.0378 (8)	0.0350 (7)	0.0002 (7)	−0.0006 (6)	−0.0010 (6)
C11	0.0302 (8)	0.0400 (8)	0.0391 (8)	−0.0041 (7)	−0.0017 (6)	0.0048 (6)
C12	0.0408 (8)	0.0382 (8)	0.0334 (7)	−0.0085 (7)	−0.0024 (6)	0.0001 (6)
C13	0.0399 (9)	0.0457 (9)	0.0407 (8)	−0.0014 (7)	0.0057 (7)	−0.0030 (7)
C14	0.0314 (8)	0.0449 (9)	0.0422 (8)	−0.0019 (7)	−0.0009 (7)	0.0017 (7)
C15	0.0513 (11)	0.0513 (10)	0.0682 (11)	−0.0078 (9)	−0.0016 (9)	−0.0219 (9)
C16	0.0497 (11)	0.0614 (12)	0.0804 (13)	−0.0063 (9)	−0.0032 (10)	−0.0139 (10)
C17	0.0362 (9)	0.0684 (12)	0.0622 (11)	0.0030 (9)	0.0043 (8)	−0.0016 (9)

### Geometric parameters (Å, °)

**Table:**

N1—C7	1.278 (2)	C5—H5A	0.93
N1—N2	1.3827 (17)	C6—H6	0.93
N2—C8	1.352 (2)	C7—H7	0.93
N2—H2	0.891 (9)	C8—C9	1.493 (2)
O1—C2	1.3589 (18)	C9—C14	1.380 (2)
O1—H1	0.82	C9—C10	1.397 (2)
O2—C3	1.3681 (19)	C10—C11	1.384 (2)
O2—C15	1.4296 (18)	C10—H10	0.93
O3—C8	1.2297 (18)	C11—C12	1.396 (2)
O4—C11	1.3703 (17)	C12—C13	1.377 (2)
O4—C17	1.422 (2)	C13—C14	1.387 (2)
O5—C12	1.3700 (17)	C13—H13	0.93
O5—H5	0.82	C14—H14	0.93
O6—H6A	0.836 (9)	C15—C16	1.498 (2)
O6—H6B	0.844 (9)	C15—H15A	0.97
C1—C2	1.392 (2)	C15—H15B	0.97
C1—C6	1.395 (2)	C16—H16A	0.96
C1—C7	1.450 (2)	C16—H16B	0.96
C2—C3	1.403 (2)	C16—H16C	0.96
C3—C4	1.378 (2)	C17—H17A	0.96
C4—C5	1.385 (2)	C17—H17B	0.96
C4—H4	0.93	C17—H17C	0.96
C5—C6	1.368 (2)		
			
C7—N1—N2	116.17 (13)	C10—C9—C8	123.32 (13)
C8—N2—N1	119.54 (13)	C11—C10—C9	120.03 (14)
C8—N2—H2	124.6 (13)	C11—C10—H10	120.0
N1—N2—H2	115.6 (13)	C9—C10—H10	120.0
C2—O1—H1	109.5	O4—C11—C10	124.89 (14)
C3—O2—C15	117.38 (12)	O4—C11—C12	114.95 (12)
C11—O4—C17	118.38 (12)	C10—C11—C12	120.16 (13)
C12—O5—H5	109.5	O5—C12—C13	122.36 (14)
H6A—O6—H6B	110.4 (17)	O5—C12—C11	118.08 (13)
C2—C1—C6	119.25 (14)	C13—C12—C11	119.53 (13)
C2—C1—C7	121.93 (14)	C12—C13—C14	120.30 (14)
C6—C1—C7	118.79 (14)	C12—C13—H13	119.8
O1—C2—C1	123.22 (13)	C14—C13—H13	119.8
O1—C2—C3	116.79 (14)	C9—C14—C13	120.67 (14)
C1—C2—C3	119.97 (14)	C9—C14—H14	119.7
O2—C3—C4	125.84 (14)	C13—C14—H14	119.7
O2—C3—C2	114.69 (13)	O2—C15—C16	107.56 (14)
C4—C3—C2	119.48 (15)	O2—C15—H15A	110.2
C3—C4—C5	120.36 (15)	C16—C15—H15A	110.2
C3—C4—H4	119.8	O2—C15—H15B	110.2
C5—C4—H4	119.8	C16—C15—H15B	110.2
C6—C5—C4	120.45 (15)	H15A—C15—H15B	108.5
C6—C5—H5A	119.8	C15—C16—H16A	109.5
C4—C5—H5A	119.8	C15—C16—H16B	109.5
C5—C6—C1	120.45 (16)	H16A—C16—H16B	109.5
C5—C6—H6	119.8	C15—C16—H16C	109.5
C1—C6—H6	119.8	H16A—C16—H16C	109.5
N1—C7—C1	121.97 (14)	H16B—C16—H16C	109.5
N1—C7—H7	119.0	O4—C17—H17A	109.5
C1—C7—H7	119.0	O4—C17—H17B	109.5
O3—C8—N2	121.72 (14)	H17A—C17—H17B	109.5
O3—C8—C9	121.55 (14)	O4—C17—H17C	109.5
N2—C8—C9	116.73 (13)	H17A—C17—H17C	109.5
C14—C9—C10	119.27 (13)	H17B—C17—H17C	109.5
C14—C9—C8	117.40 (13)		

### Hydrogen-bond geometry (Å, °)

**Table:**

*D*—H···*A*	*D*—H	H···*A*	*D*···*A*	*D*—H···*A*
O1—H1···N1	0.82	1.94	2.6529 (16)	144
O5—H5···O6^i^	0.82	1.81	2.6177 (17)	170
O6—H6A···O3^ii^	0.84 (1)	1.96 (1)	2.7908 (17)	176 (2)
O6—H6B···O1^iii^	0.84 (1)	2.08 (1)	2.8714 (18)	157 (2)
N2—H2···O4^iv^	0.89 (1)	2.54 (2)	3.1181 (17)	123 (2)
N2—H2···O5^iv^	0.89 (1)	2.19 (1)	3.0496 (18)	163 (2)

Symmetry codes: (i) *x*−1, *y*, *z*; (ii) *x*+1/2, −*y*+1/2, *z*−1/2; (iii) −*x*+3/2, *y*−1/2, −*z*+3/2; (iv) −*x*−1/2, *y*+1/2, −*z*+3/2.
